# Late Diagnosis of Primary Hyperoxaluria in an Adult Patient With End-Stage Renal Disease and Bicytopenia

**DOI:** 10.7759/cureus.42469

**Published:** 2023-07-26

**Authors:** Achref Miry, Mohammed Tbouda, Youssef Bouhajeb, Sanae Abbaoui

**Affiliations:** 1 Pathology, Faculty of Medicine and Pharmacy of Agadir, Agadir, MAR; 2 Pathology, Souss Massa University Hospital, Agadir, MAR; 3 Pathology, Mohammed V Military Hospital, Agadir, MAR; 4 Pathology, Ibn Rochd Pathology Center, Agadir, MAR

**Keywords:** bone marrow failure, renal failure, primary hyperoxaluria, bicytopenia, bone marrow, oxaluria

## Abstract

Primary hyperoxaluria (PH) is a rare genetic condition that disrupts the normal process of glyoxylate metabolism, resulting in an overproduction of oxalate. This excessive oxalate production leads to the accumulation of calcium oxalate (known as oxalosis) throughout various organs in the body. The urinary tract, specifically the renal parenchyma, is the first location where the deposition of calcium oxalate begins in PH. These deposits are responsible for nephrocalcinosis and tubule‑interstitial nephritis which leads to end‑stage renal failure. This is then followed by the accumulation of oxalate in other organs including the bone marrow. Herein, we report the case of a 22-year-old male patient who presented with bicytopenia; he had a history of end-stage renal disease preceded by recurrent urolithiasis and nephrolithiasis episodes since the age of 3 years. A bone marrow biopsy was performed for evaluation of the bicytopenia which led to the diagnosis of PH.

## Introduction

Hyperoxaluria is a condition where the levels of oxalate in the blood and urine (oxaluria) are elevated. Primary hyperoxaluria (PH) is a rare autosomal recessive genetic metabolic disorder that occurs in the pathway of glyoxylate metabolism, resulting in the excessive production of oxalate [1,2]. This condition is characterized by the deposition of calcium oxalate throughout numerous organs in the body [3]. On the other hand, secondary hyperoxaluria (SH) is an acquired disorder that occurs because of factors like excessive intake of oxalate through diet, Crohn's disease, chronic hemodialysis, or bowel resection. Before diagnosing PH, it is important to exclude these potential causes [3]. The combination of oxalate with calcium has a strong tendency to accumulate in numerous organs [4]. This condition is called oxalosis and involves the deposition of calcium oxalate crystals in the kidneys and other organs [5]. Initially, the tubulointerstitium of the renal parenchyma is the primary site for the deposition of calcium oxalate, leading to acute and chronic tubulointerstitial nephritis, nephrolithiasis, and subsequent renal failure. Following the deposition in the kidneys, calcium oxalate crystals also accumulate in the bone marrow and other tissues. The widespread replacement of the bone marrow tissue by these crystals results in a range of hematologic manifestations [2,6], namely pancytopenia and leukoerythroblastic reaction [7]. We report the case of a 22-year-old male patient with end-stage renal disease and bicytopenia, in whom the diagnosis of PH was based on a bone marrow biopsy performed for evaluation of the bicytopenia and which revealed a widespread deposition of oxalate crystals.

## Case presentation

We report the case of a 22-year-old male patient, with an eight-year history of end-stage renal disease for which he had been receiving hemodialysis three times a week. The end-stage renal disease in our patient was preceded by recurrent urolithiasis and nephrolithiasis episodes since the age of 3 years. He also reported chronic bilateral knee and elbow pain. Our patient presented for the workup of a bicytopenia. No digestive symptoms suggestive of Crohn’s disease were found in our patient’s history, he also had no excessive oxalate intake. Physical examination revealed moderate splenomegaly. The patient's complete blood count showed a hemoglobin level of 7.9 g/dL, a white blood cell (WBC) count of 3.5 × 109/L, and a normal platelet count of 170×109/L. The creatinine level was 7mg/dL and the blood urea nitrogen (BUN) level was 54 mg/dL. The ferritin level was 1990 ng/mL. The potassium, bilirubin, and liver enzymes were within the normal range. Hepatitis B virus, hepatitis C virus, Epstein-Barr virus (EBV), and HIV serologies were performed and were negative (Table 1).

**Table 1:** Summary of laboratory parameters in our patient

Parameter	Results	Normal range and unit
Hemoglobin	7.9 g/dl	12-18 g/dl
White blood cell	3.5 × 10^9 ^elements/L	5-9 x 10^9^ elements/L
Platelets	170×10^9 ^elements/L	150-450×10^9^ elements/L
Serum creatinine	7 mg/dL	0.7-1.3 mg/dl
Blood urea nitrogen (BUN)	54 mg/dL	7-20 mg/dL
Ferritin	1990 ng/mL	25-200 ng/mL
Serum oxalate	325 micrograms/dL	<45 micrograms/dL

**Figure 1:**
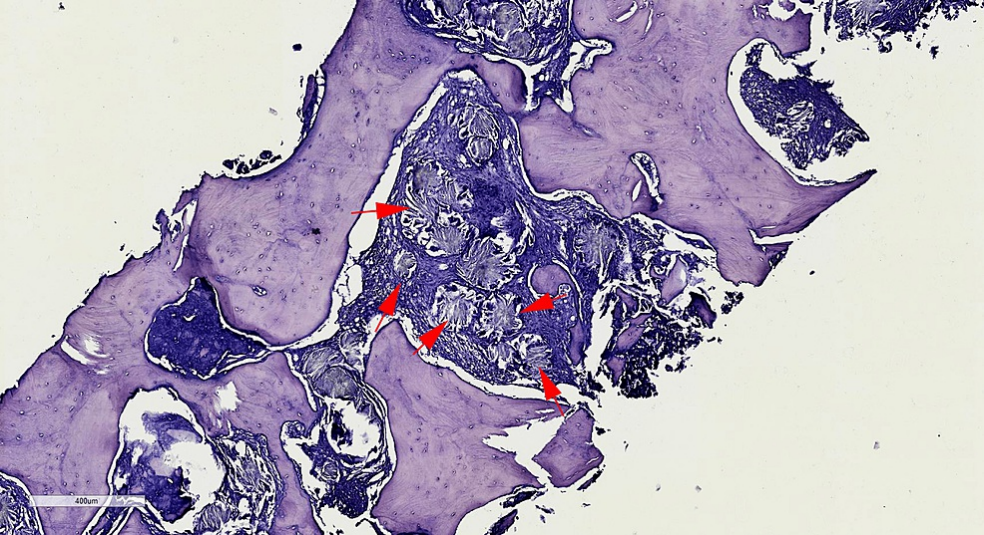
Microphotography showing a fibrotic bone marrow tissue containing numerous rosette-shaped calcium oxalate crystals (red arrows) (H&E, 40x)

**Figure 2:**
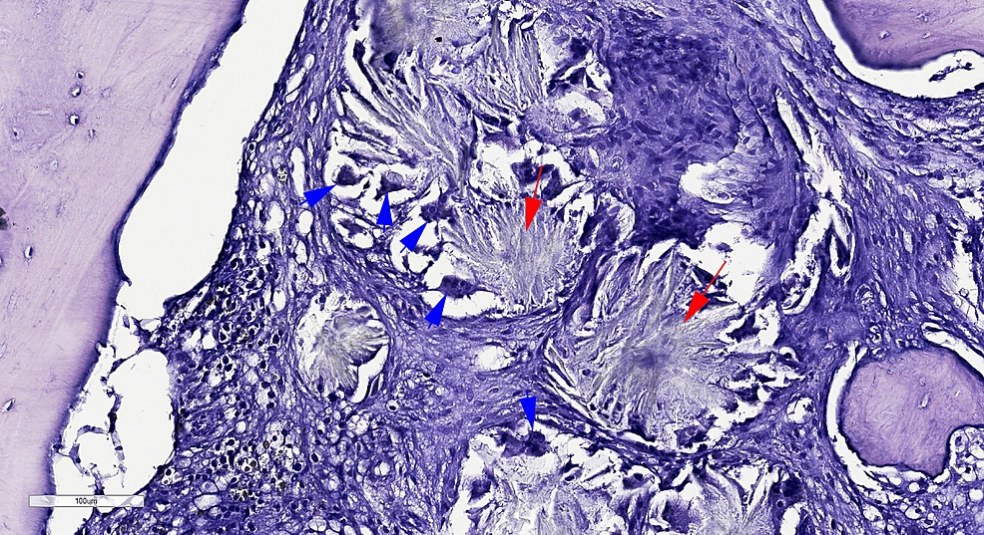
Microphotography showing needle-shaped oxalate crystals (red arrows), surrounded by foreign body giant cells (blue arrows) (H&E, 100x)

Abdominal ultrasonography was performed revealing diffuse nephrolithiasis and hydronephrosis. For the evaluation of the bicytopenia, a bone marrow trephine biopsy was performed. Pathological assessment of the bone marrow biopsy revealed a fibrotic bone marrow tissue containing numerous rosette-shaped deposits of needle-shaped oxalate crystals that were often surrounded by foreign body giant cells (Figures 1-2). Only rare scattered hematopoietic cells could be observed.

The dosage of serum oxalate was performed after the bone marrow biopsy findings. The serum oxalate level was 325 micrograms/dL. In our case, the morphology of the observed bone marrow crystals, the absence of secondary causes of oxaluria, and the primary renal involvement helped to direct the diagnosis toward PH. This diagnosis was confirmed after the identification of c830C>A p and Ala277Asp missense mutations on exon 8 of AGXT gene. Our patient is a candidate for simultaneous liver and kidney transplantation.

## Discussion

In the case of our patient, a bone marrow biopsy was performed for evaluation of the bicytopenia. The findings of the bone marrow biopsy were highly suggestive of PH which indicated an assessment of the mutational status of the AGXT gene. This enabled the confirmation of the diagnosis of PH. In the literature, the diagnosis of PH based on pathological findings in bone marrow biopsy is uncommon [7].

PH is an uncommon genetic disorder affecting the glyoxylate metabolism pathway, resulting in excessive oxalate production and its accumulation as calcium oxalate in various organs. PH is classified into three types, all of which are inherited in an autosomal recessive manner. Type I PH accounts for approximately 70% of the cases. This particular type is caused by a deficiency in an enzyme called alanine glyoxylate aminotransferase, responsible for converting glyoxylate into glycine. Type I PH exhibits significant variability in its clinical manifestations. The age of onset can range from infancy to over 50 years old. Approximately 10% of PH cases fall under the category of type II disease, which arises from a deficiency in the glyoxylate reductase/hydroxypyruvate reductase (GRHPR) enzyme. This enzyme is responsible for converting glyoxylate into glycolate. Type II PH is generally less severe compared to type I PH, exhibiting milder symptoms and later onset of initial presentations [8]. Type III PH encompasses approximately 10% of PH cases [9]. This specific type results from a defect in 4-hydroxy-2-oxoglutarate aldolase, an enzyme responsible for breaking down 4-hydroxy-2-oxoglutarate into pyruvate and glyoxylate. The average age of onset is around 2 years, and common manifestations such as pain, hematuria, and urinary tract infections are primarily attributed to urolithiasis. Compared to the other types of PH, type III exhibits the mildest symptoms and does not progress to end-stage renal disease [10].

The renal parenchyma is the primary organ where calcium oxalate deposits occur. This deposition is a result of the oversaturation of urine with oxalate, leading to the aggregation of crystals, urolithiasis, and/or nephrolithiasis. Persistent nephrolithiasis can ultimately progress to end-stage renal disease. Furthermore, calcium oxalate deposits may also accumulate in other organs except for the liver, including the retina, central nervous system, myocardium, skin, and bone marrow [8,11]. The deposition of oxalate crystals in the bone marrow is an infrequent occurrence, giving rise to diverse hematologic manifestations. These include anemia that is unresponsive to erythropoietin, myelophthisic anemia, and pancytopenia resulting from the substitution of the marrow by oxalate crystals [2,6,11].

The onset of symptoms in type I PH typically occurs around the median age of 5 years, with initial manifestations predominantly associated with involvement of the urinary tract [12]. Type I PH can manifest with various renal presentations at different stages of life, such as nephrocalcinosis and renal failure during infancy, recurrent urolithiasis leading to renal failure during childhood or adolescence, recurrence of renal failure after transplantation, or a presymptomatic status with a family history of PH [13].

Timely identification of individuals with PH is crucial for better long-term prognosis [14]. Regrettably, the diagnosis of PH is frequently delayed as in our reported case. Nonetheless, there are several tests and procedures available to identify potentially affected patients. Stone analysis, measurement of urine oxalate levels, and determination of plasma oxalate concentration can provide valuable insights. To establish a definitive diagnosis of PH, liver biopsy assessment, measurement of enzyme activity, and DNA testing for the presence of mutated genes are performed. In cases where there is a suspicion of PH based on family history, genetic counseling should be considered. Prenatal diagnosis can be achieved by analyzing DNA obtained from chorionic villous biopsy samples [15].

In our case, the diagnosis was based on bone marrow biopsy findings and confirmed by analysis of the mutational status of the AGXT gene. It is worth noting that the rise of serum oxalate levels is not synonymous with oxaluria and can also be observed in end-stage renal disease or patients undergoing hemodialysis [16]. There is limited literature available in English documenting cases of bone marrow oxalosis, often accompanied by varying degrees of cytopenias, leukoerythroblastic reaction, and resistance to erythropoietin [16]. Pancytopenia resulting from the deposition of oxalate crystals in the bone marrow is a rare complication associated with PH [16].

On the therapeutic level, in order to enhance the quality of life and delay the need for kidney transplantation, medical recommendations and treatments are implemented for individuals with PH. These interventions include consuming large volumes of fluids, restricting the consumption of foods high in oxalate, administering pyridoxine to facilitate the conversion of glyoxylate to glycine, and undergoing periodic dialysis to lower oxalate levels [16]. However, it is important to note that dietary restrictions may not be equally significant for all individuals with PH and are primarily recommended for secondary cases [16]. This highlights the critical role of early diagnosis as a fundamental requirement for successful treatment outcomes [16]. Combined liver and kidney transplantation is considered a potential curative treatment for PH. Liver transplantation is particularly effective in correcting the underlying enzyme deficiency [16]. It is important to note that isolated kidney transplantation is often ineffective for most patients due to the continued presence of oxalate overload, which frequently results in allograft loss. This phenomenon of graft loss in patients with PH was reported in many case reports [16]. In the case of pancytopenia secondary to PH, as in our case, the reversal of this complication is exceptionally rare after transplantation. However, Sud et al. documented a case in which pancytopenia, resulting from the infiltration of oxalate crystals in the bone marrow, was successfully reversed following a kidney transplant alone [6]. As bone marrow involvement in PH is rare, the resolution of cytopenia following transplantation remains a subject of controversy.

## Conclusions

Despite its rarity, PH should be considered as a potential underlying cause in infants and children presenting with nephrolithiasis, as well as in adult patients with recurrent nephrolithiasis episodes. Early diagnosis and appropriate treatment play a crucial role in preventing subsequent complications, including the development of bone marrow oxalosis which can lead to marrow failure. As bone marrow involvement in PH is rare, the resolution of cytopenia following transplantation remains a subject of controversy.

## References

[1] Harambat J, Fargue S, Acquaviva C (2010). Genotype-phenotype correlation in primary hyperoxaluria type 1: the p.Gly170Arg AGXT mutation is associated with a better outcome. Kidney Int.

[2] Mykytiv V, Campoy Garcia F (2018). Anemia in patient with primary hyperoxaluria and bone marrow involvement by oxalate crystals. Hematol Oncol Stem Cell Ther.

[3] Farhi DC, Chai CC (2009). Pathology of Bone Marrow and Blood Cells.

[4] Beers MH, Albert R (2006). The Merck Manual of Diagnosis and Therapy.

[5] Hassan K, Qaisrani JH, Qazi H, Naseem L, Zaheer HA, Zafar T (2006). Oxalosis in the bone and bone marrow. Int J Pathol.

[6] Sud K, Swaminathan S, Varma N, Kohli HS, Jha V, Gupta KL, Sakhuja V (2004). Reversal of pancytopenia following kidney transplantation in a patient of primary hyperoxaluria with bone marrow involvement. Nephrology (Carlton).

[7] Hassan MN, Abd Rahman WSW, Zulkafli Z (2014). Bone marrow involvement in systemic oxalosis presenting as pancytopenia and uremia—a first reported case in Malay population. Research.

[8] Hoppe B, Langman CB (2003). A United States survey on diagnosis, treatment, and outcome of primary hyperoxaluria. Pediatr Nephrol.

[9] Milliner DS, Harris PC, Sas DJ, Lieske JC (1993). Primary Hyperoxaluria Type 3. https://www.ncbi.nlm.nih.gov/books/NBK316514/.

[10] Belostotsky R, Seboun E, Idelson GH (2010). Mutations in DHDPSL are responsible for primary hyperoxaluria type III. Am J Hum Genet.

[11] Nematollahi P, Mohammadizadeh F (2015). Primary hyperoxaluria diagnosed based on bone marrow biopsy in pancytopenic adult with end stage renal disease. Case Rep Hematol.

[12] Cochat P, Deloraine A, Rotily M, Olive F, Liponski I, Deries N (1995). Epidemiology of primary hyperoxaluria type 1. Nephrol Dial Transplant.

[13] Sharifian M, Yeganeh MH, Rouhipour A, Jadali F, Gharib A (2012). Photoclinic. Arch Iran Med.

[14] Kopp N, Leumann E (1995). Changing pattern of primary hyperoxaluria in Switzerland. Nephrol Dial Transplant.

[15] Hoppe B, Beck BB, Milliner DS (2009). The primary hyperoxalurias. Kidney Int.

[16] Nematollahi P, Mohammadizadeh F (2015). Primary hyperoxaluria diagnosed based on bone marrow biopsy in pancytopenic adult with end stage renal. Case Rep Hematol.

